# Experience of stigmatization in children receiving inpatient and outpatient mental health treatment: a longitudinal study

**DOI:** 10.1007/s00787-021-01904-5

**Published:** 2021-11-09

**Authors:** Anya Kaushik, Efstathios Papachristou, Laurence Telesia, Danai Dima, Sandra Fewings, Evgenia Kostaki, Jorge Gaete, George B. Ploubidis, Marinos Kyriakopoulos

**Affiliations:** 1 National and Specialist Acorn Lodge Inpatient Children Unit, South London and Maudsley NHS Foundation Trust, London, UK; 2 Department of Psychology and Human Development, UCL Institute of Education, University College London, London, UK; 3 Department of Child and Adolescent Psychiatry, Institute of Psychiatry, Psychology and Neuroscience (PO66), King’s College London, De Crespigny Park, London, SE5 8AF UK; 4 Department of Psychology, School of Arts and Social Sciences, City, University of London, London, UK; 5 Department of Neuroimaging, Institute of Psychiatry, Psychology and Neuroscience, King’s College London, London, UK; 6 Learning Disability Specialist Health Services, Hertfordshire Partnership University NHS Foundation Trust, Braintree, Essex UK; 7 Faculty of Education, Universidad de los Andes, Santiago, Chile; 8 Millennium Nucleus To Improve the Mental Health of Adolescents and Youths, Santiago, Chile; 9 Centre for Longitudinal Studies, UCL Social Research Institute, University College London, London, UK; 10 First Department of Psychiatry, National and Kapodistrian University of Athens Medical School, Eginition Hospital, Athens, Greece

**Keywords:** Stigma, Children, Mental health treatment, Inpatient, Outpatient, Global functioning

## Abstract

Mental health-related stigma is poorly understood, and minimal research has focused on the experience of stigma from children’s perspectives. We sought to investigate whether children treated as inpatients and outpatients had different experiences of stigma over time and whether stigma is linked to global functioning cross-sectionally and longitudinally. Children, aged 8–12 years, receiving treatment within a national specialist mental health inpatient unit were matched for age, gender and diagnosis with children receiving outpatient treatment (*N* = 64). Validated measures of stigma, global functioning and symptom severity were collected at the start of treatment and upon discharge from the ward for inpatients, and a similar timeframe for their individually matched outpatients. Latent change score models and partial correlation coefficients were employed to test our hypotheses. No differences in most aspects of stigma between children treated as inpatients and outpatients were observed, except for personal rejection at baseline and self-stigma at follow-up favouring outpatients. A reduction in stigma was observed in societal devaluation, personal rejection and secrecy for inpatients, and self-stigma and secrecy for outpatients between the two assessments. Societal devaluation declined at a higher rate among inpatients compared to outpatients, albeit reductions in stigma were comparable for all remaining measures. No association was found between the change in stigma and change in global functioning. Future research may offer further insights into the development and maintenance of stigma and identify key targets for anti-stigma interventions to reduce its long-term impact.

## Introduction

Mental illness generates a substantial disease burden across the world [1, 2], with its prevalence seeming to be on the rise [3]. This appears to be evident in both adults [4] and young people [5]. Half of adult mental health problems start before 15 years of age, almost 75% start before 18 years of age, and most of these conditions are undetected and untreated [6]. Mental health difficulties in early life have been associated with less favourable outcomes in the long-term and elevated risk of premature mortality, suggesting that effective early interventions may improve not only mental wellbeing but also physical health at a population level [7]. Despite the availability of effective treatments [8, 9], only a quarter of young people with mental health difficulties have had contact with appropriate services [5]. The stigma associated with these difficulties has been identified as an important factor preventing access to treatment for both young people [10] and their parents [11].

Stigma is the process by which negative stereotypes and prejudices lead to discrimination against individuals with certain characteristics [12]. The stigmatisation of people with mental illness has been well established as a complex global problem [13]. Discrimination may take the form of avoiding social contact with stigmatized individuals or limiting individuals’ access to employment and housing [12]. Those holding stigmatising views are also less likely to seek mental health support themselves [14].

The stigma of mental illness in young people has not been studied as extensively as in adults [15]. There are considerable differences between adults and young people which are likely to contribute to different experiences of mental health-related stigma. Depending on their stage of development, children may not have the situational awareness to understand that they are stigmatised by others, nor to internalise this into self-stigma. Furthermore, decisions about accessing mental health support are typically moderated by their parents or caregivers, which is usually not the case for adults. Therefore, the degree to which findings from the adult studies can be extrapolated to the field of child and adolescent psychiatry is uncertain [16].

As is the case within the adult mental health literature [17], much of the research pertaining to stigma in young people has focused on public stigma (i.e. participants recruited from within non-clinical populations), with minimal investigation of stigmatised individuals’ experience of stigma [15, 18]. Qualitative studies allow researchers to explore the experiences of young people with mental illness [19], although robust direct comparisons between groups is often not possible with these methods. Quantitative measures of young people’s experiences of stigma have been validated and used in adolescents [20] and more recently children [21, 22].

The impact of psychiatric hospital admissions on stigma has long been recognised as an important field of study [23]. There has been criticism of psychiatric hospitals dating back to the 1960s and 70s, with concerns that they had the potential to increase stigma [24]. Whilst there has been a largescale movement towards the deinstitutionalisation of psychiatric care, questions continue to be raised about the psychosocial impact of psychiatric hospitals and to ensure compliance, and the experience of coercion has been associated with greater stigma towards psychiatric admission [27]. Furthermore, many patients feel they have been treated differently after a psychiatric admission [28].

To the authors’ knowledge, no studies to date have investigated whether there is a difference in stigma experiences between children (aged 8–12 years) treated within an inpatient vs. outpatient mental health setting. Inpatient treatment in children may not be as stigmatising as in adults, considering its links with hospital education and the fact that residential educational settings are an acceptable option in this age group. In addition, within adults, hospital admissions are likely to be associated with more severe mental illness, which may attract stigma in itself. With young people, the association may be less clear-cut; neurodevelopmental, social, cultural, and family factors may play a larger part in the decision of whether to offer inpatient or outpatient treatment. Finally, the way the functional impairment experienced by children in need of hospital admission and the improvements following this affect stigmatisation may be different compared to adolescents or adults due to their developmental stage.

The current study aimed to explore the experience of stigmatisation in children receiving mental health treatment as inpatients comparatively to outpatients over time. We hypothesised that:

1. There will be no difference in the experience of stigma between children treated for mental illness within an inpatient vs. outpatient setting,
2. Stigma will be linked to functional impairment, with improvements in function associated with reduced stigma.

## Methods

### Procedure

Children were identified to be recruited into one of two groups, outpatient or inpatient, dependent on the type of mental health treatment they were receiving. Once potential participants were identified, they and their parents/carers were provided with written information about the study before deciding to enrol. Written informed consent from parents/carers and written informed assent from children were obtained.

As part of a wider study to validate the Paediatric Self-Stigmatization Scale (PaedS), a total sample of 156 children aged 8–12 years receiving mental health treatment by NHS Child and Adolescent Mental Health Services (CAMHS) were recruited. Of these, 37 were receiving inpatient treatment in a national 10-bed children’s mental health unit, and 119 were treated as outpatients. Details for all participants can be found in a previous publication [21].

For the purposes of the present study, 32 children receiving inpatient treatment were matched individually, as closely as possible, for age, gender and primary diagnosis with children receiving outpatient treatment, resulting in a total sample size of 64 children. The children receiving inpatient treatment and their parents/carers were asked to complete the study questionnaires as close as possible to their admission date, and again as close as possible to discharge from the inpatient unit (time between assessments in days *M* = 101.41; SD = 10.70). Those receiving outpatient treatment, and their parents/carers, were asked to complete the study questionnaires during their treatment and a second time within a similar timeframe to their matched inpatient peers (time between assessments in days *M* = 168.56; SD = 18.29).

The study was approved by the National Research Ethics Service 102 Committee South East Coast—Kent.

### Measures

Paediatric Self-Stigmatization Scale (PaedS). This is a recently validated child-specific measure of stigma [21]. It consists of 4 subscales that measure societal devaluation (14 items), personal rejection (5 items), self-stigma (5 items) and secrecy of receiving mental health treatment (7 items). All subscales, apart from the personal rejection subscale, are scored using a 4-point Likert scale in which higher scores indicate greater stigmatization. The personal rejection subscale contains items for which the child is requested to give a positive or a negative answer (Yes = 1, No = 0). The PaedS takes around 5–10 min to complete. The internal consistency is high for all subscales, ranging from α = 0.72 (personal rejection subscale) to α = 0.86 (societal devaluation subscale) [21].

Children’s Global Assessment Scale (CGAS) [29]. This scale measures global functioning and has values from 1, representing the lowest level of functioning, to 100, representing the highest. Scores over 70 represent normal functioning. The CGAS, administered by a trained clinician, has good interrater reliability (0.84) and test–retest stability (0.85) [29] and has been used extensively in clinical and research settings.

Strengths and Difficulties Questionnaire (SDQ) [30], parent version. This questionnaire has 25 items, divided in five subscales: emotional symptoms, conduct problems, hyperactivity/inattention, peer relationship problems and prosocial behaviour. Each item uses a three-point ordinal format to be answered with: 0 = not true; 1 = somewhat true; and 2 = certainly true. We only report the scores of the emotional symptoms, conduct problems, hyperactivity/inattention, and peer relationship problems subscales in this study. Higher scores indicate greater psychological dysfunction.

Pediatric Quality of Life Inventory version 4.0 [31]. This scale consists of four subscales (physical, emotional, social and school functioning) of 23 items in total scored on a 5-point Likert scale. Scores can range from “Never” to “Almost always”, with a higher score indicating better quality of life. The version of this scale relevant to children aged 8–12 years was used in this study.

### Statistical analysis

Descriptive statistics were used to present the sociodemographic and clinical features of the sample. We used a series of *t* tests to compare inpatients and outpatients at baseline and follow-up for differences in the mean scores on the PaedS scales, CGAS, parent-reported SDQ subscales and self-reported quality of life scores.

Next, we ran univariate Latent Change Score (LCS) models—also known as latent difference or Difference in Differences (DID) models—for each of the four PaedS subscales and the CGAS to identify changes in scores between the baseline and the follow-up. LCS models are a powerful class of Structural Equation Models (SEM) which are typically used to replicate *t* tests or repeated measures ANOVA because they do not rely on the assumptions of more traditional statistical tests, such as the assumptions of normal distribution and sphericity. They also provide added flexibility in handling missing data and allow for the modelling of within and between person variations for the score differences [32–34]. In an LCS framework, post-scores are regressed on the baseline scores and the path is fixed to 1.0. A latent variable is then defined that loads on the post-scores with its path also fixed to 1.0. Using this parameterisation, the autoregressive paths specify that the time elapsed between T1 and T2 in the ‘control’ and ‘experimental’ group is equitemporal and is analogous to the parallel trends assumption on which more traditional DID models rely. The latent construct captures the difference between pre- and post-scores while estimating a mean and a variance for the latent difference.

For our study we ran a series of univariate LCS models on the complete sample as well as after stratifying by patient type (inpatients and outpatients). In light of differences in the duration between the baseline and follow-up assessments, all LCS models were adjusted for the time interval (in days) between the two assessments. Score changes were compared between inpatients and outpatients by means of Wald tests, using equality constraints in the univariate multi-group LCS models. After fitting the univariate LCS models, we aimed to test the correlations between the latent difference scores of these univariate models.

**Fig. 1:**
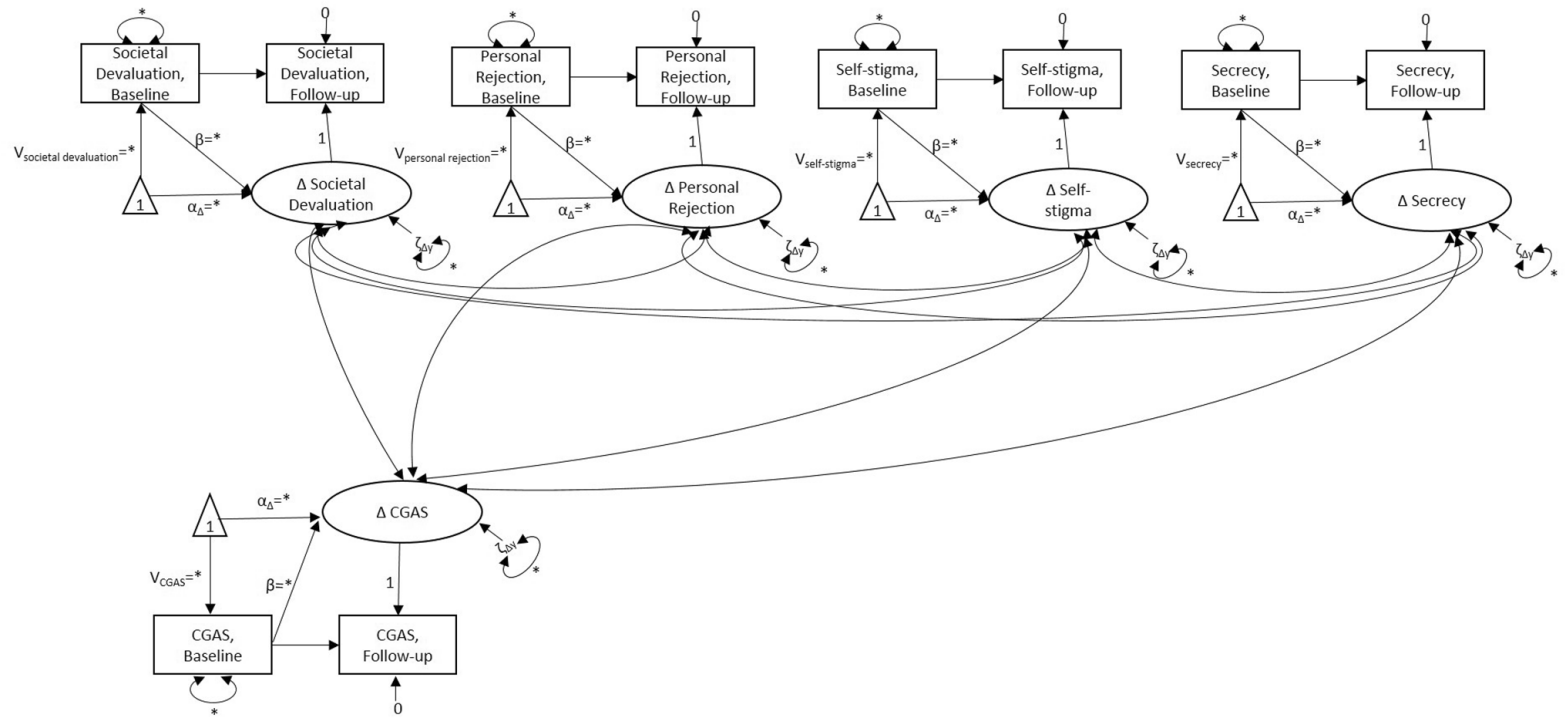
Latent change score (difference in differences) model examining the parallel change in scores of the self-reported scales Paediatric Self-Stigmatization Scale (PaedS) and Children’s Global Assessment Scale (CGAS) (squares = observed variables; circles = latent variables; rectangles = constants; single-headed arrows = regression paths; bi-headed arrows = correlations). Correlations between individual PaedS and CGAS scores in this study were examined using the latent difference scores of univariate latent change score models as observed variables in a two-step approach

The proposed analytical model is illustrated in Fig. 1. Two-headed arrows between the latent change scores represent the correlations between the latent constructs. Due to the complexity of the proposed model and the limited sample size, we used a two-step approach for the estimation of the correlations between the latent difference scores, whereby the latent difference scores calculated in the univariate LCS were extracted and used as manifest (observed) variables for further testing.

Partial correlation coefficients were calculated to examine the associations between the latent difference scores of the PaedS subscales and the CGAS after adjusting for the time elapsed between the two assessments. A final set of analyses included the examination of the predictive ability of self-reported quality of life and parental reported SDQ for the score differences computed in the univariate LCS. Linear regression models were run, for which self-reported quality of life and parental-reported total SDQ scores were used as predictors of the difference scores of each the PaedS subscales and the CGAS.

LCS were performed in MPlus 7.4 [35] using maximum likelihood estimation with robust standard errors (MLR) which can account for skewed distributions of data. Missing data on the outcomes were handled using full information maximum likelihood where appropriate, i.e. when a participant had missing data for one of the two assessments (baseline or follow-up). All remaining analyses were performed in Stata/SE 15 [36].

## Results

The analytic sample comprised 64 children, 32 (50%) of whom were inpatients. Of these children, 22 had diagnoses of emotional/behavioural/eating disorders and combinations (mood and anxiety disorders, obsessive compulsive disorder, dissociative and somatoform disorders, post-traumatic stress and adjustment disorders, conduct disorders, eating disorders), 22 had diagnoses of neurodevelopmental disorders and combinations (autism spectrum disorders, attention deficit hyperactivity disorders, tic disorders, psychotic disorders, intellectual disability, specific developmental disorders), and 20 had a combination of the above categories. Table 1 presents clinical and sociodemographic variables at baseline and follow-up, stratified by patient group. At baseline, inpatients scored higher on the personal rejection subscale of the PaedS (*p* = 0.04) and scored significantly lower in the CGAS (*p* < 0.001) when compared to outpatients. At follow-up, inpatients scored significantly higher in the self-stigma subscale of the PaedS (*p* = 0.01), while outpatients scored higher in the emotional problems’ subscale (*p* = 0.04) and the hyperactivity subscale (*p* = 0.001) of the SDQ. Finally, the time interval (in days) between the baseline and follow-up assessments for outpatients was, on average, 67.15 days longer than inpatients (*p* = 0.002). The two groups did not differ significantly with respect to the remaining PaedS and SDQ scale scores, their mean age and sex distribution, or their parental-reported quality of life at either timepoint.

**Table 1:** Baseline and outcome characteristics

	Inpatients (*N* = 32)	Outpatients (*N* = 32)	*p* values
Sex [*n* (%) male]	17 (53%)	18 (56%)	0.80
Age	10.84 (0.23)	10.59 (0.27)	0.48
T1–T2 time interval (in days)	101.41 (10.70)	168.56 (18.29)	0.002
Baseline			
PaedS^a^ scores			
Societal devaluation	2.39 (0.10)	2.34 (0.13)	0.76
Personal rejection	0.45 (0.06)	0.28 (0.06)	0.04
Self-stigma	2.37 (0.16)	2.10 (0.16)	0.23
Secrecy	2.94 (0.11)	2.78 (0.14)	0.38
CGAS^b^	37.39 (2.94)	60.48 (2.09)	< 0.001
Parental-reported SDQ^c^ scores			
Emotional problems	6.06 (0.44)	6.23 (0.49)	0.81
Peer problems	4.03 (0.46)	3.97 (0.44)	0.92
Conduct problems	3.58 (0.47)	3.53 (0.41)	0.94
Hyperactivity problems	5.68 (2.76)	6.69 (0.43)	0.13
Self-reported PEDSQL score	55.46 (3.25)	59.04 (3.72)	0.47
Follow-up			
PaedS scores			
Societal devaluation	2.26 (0.13)	2.20 (0.11)	0.69
Personal rejection	0.28 (0.06)	0.21 (0.04)	0.33
Self-stigma	2.27 (0.15)	1.82 (0.13)	0.01
Secrecy	2.73 (0.11)	2.48 (0.13)	0.13
CGAS	61.63 (2.32)	62.63 (2.29)	0.76
Parental-reported SDQ scores			
Emotional problems	4.68 (0.46)	6.16 (0.52)	0.04
Peer problems	3.07 (0.44)	3.56 (0.38)	0.39
Conduct problems	2.84 (0.48)	3.53 (0.43)	0.29
Hyperactivity problems	4.45 (0.60)	6.91 (0.42)	0.001
Self-reported PEDSQL score	66.98 (2.68)	59.97 (3.15)	0.10

Values expressed as M (SE) unless otherwise specified

*PaedS* Paediatric Self-Stigmatization Scale, *CGAS* Children’s Global Assessment Scale, *SDQ* Strengths and Difficulties Questionnaire, *PEDSQL* Pediatric Quality of Life Inventory version 4.0

Table 2 presents results of the univariate LCS models. The results of the combined sample analysis suggest that patients as a whole group showed improved functioning (higher CGAS scores) and also reduced scores on the personal rejection, self-stigma and secrecy subscales of the PaedS following treatment. After stratifying the sample by patient type (inpatients and outpatients), the results showed that inpatients scored lower on the societal devaluation, personal rejection, and secrecy subscales of the PaedS, and also showed improved functioning over time. Outpatients reported lower levels on the self-stigma and secrecy subscales at follow-up, while also showing a marginal yet statistically significant improvement in their CGAS scores (*p* = 0.03).

**Table 2:** Latent score differences between baseline and follow-up assessments in self-reported PaedS and CGAS scores

	Combined		Inpatients		Outpatients		Wald test comparing Δ scores between inpatients and outpatients
	Mean (SE) difference (Δ)	Variance of Δ (SE)	Mean (SE) difference (Δ)	Variance of Δ (SE)	Mean (SE) difference (Δ)	Variance of Δ (SE)	
Self-reported PaedS scores							
Societal devaluation	− 0.11 (0.05)*	0.13 (0.03)**	− 0.15 (0.06)**	0.07 (0.02)**	− 0.06 (0.09)	0.14 (0.04)**	9.72 (1), *p* = 0.002
Personal rejection	− 0.11 (0.04)**	0.09 (0.02)**	− 0.16 (0.06)**	0.10 (0.02)**	− 0.06 (0.05)	0.07 (0.02)**	1.28 (1), *p* = 0.26
Self-stigma	− 0.19 (0.09)*	0.57 (0.11)**	− 0.10 (0.14)	0.57 (0.14)**	− 0.28 (0.13)*	0.51 (0.13)**	0.28 (1), *P* = 0.60
Secrecy	− 0.26 (0.07)**	0.31 (0.05)**	− 0.22 (0.10)*	0.28 (0.06)**	− 0.28 (0.11)*	0.32 (0.09)**	1.52 (1), *p* = 0.22
CGAS^b^	13.63 (2.21)**	285.27 (66.63)**	24.16 (3.15)**	303.32 (79.57)**	2.42 (1.08)*	32.03 (14.65)*	9.59 (1), *p* = 0.002

*PaedS* Paediatric Self-Stigmatization Scale, *CGAS* Children’s Global Assessment Scale

*p < 0.05; **p < 0.001

A series of Wald tests comparing the mean latent difference scores between the two groups showed that the improvement observed for the CGAS and the PaedS societal devaluation scores in inpatients was significantly higher compared to outpatients (*p* = 0.002 for both tests). The improvements observed on the remaining PaedS subscales, including personal rejection, self-stigma and secrecy were small in magnitude between the two patient groups and did not reach statistical significance (all *p* values > 0.05). Noticeably, the variance estimates for the score differences were statistically significant for all four PaedS subscale- and CGAS scores, suggesting significant inter-individual variability in improvement between the two assessments.

Next, we ran a series of partial correlations to examine which of the PaedS subscale scores improved in parallel and also whether improvements in CGAS were associated with reduced levels of stigmatization (Table 3). Overall, improvements on the societal devaluation subscale were significantly associated with decreased scores on the personal-rejection (*p* < 0.001) and self-stigma subscales (*p* = 0.02). Changes in the secrecy subscale were also significantly correlated with those in the self-stigma subscale (*p* = 0.003). Among inpatients, the societal devaluation and personal rejection subscales of the PaedS increased in parallel (*p* = 0.002). Among outpatients, three pairwise-associations between the PaedS subscales showed contemporaneous improvements including the following: societal devaluation-personal rejection (*p* = 0.04), societal devaluation- self-stigma (*p* = 0.01), and self-stigma- secrecy (*p* = 0.01). Moreover, improvements in CGAS scores were significantly associated with changes in the personal rejection subscale (*p* = 0.05) in outpatients. However, no other significant associations were found between changes in CGAS scores and changes any of the remaining PaedS subscales in the combined sample analysis or any of the groups independently.

**Table 3:** Partial correlation coefficients of score differences between self-reported PaedS and CGAS scores adjusted for the time interval elapsed between baseline and follow-up assessments

	Combined					Inpatients					Outpatients				
	**1**	**2**	3	4	5	1	2	3	4	5	1	2	3	4	5
1. Societal devaluation	–					–					–				
2. Personal rejection	0.48**	–				0.54**	–				0.37*	–			
3. Self-stigma	0.29*	0.20	–			0.18	0.25	–			0.45*	0.27	–		
4. Secrecy	0.07	0.07	0.37**	–		− 0.18	− 0.12	0.26	–		0.23	0.33	0.45*	–	
5. CGAS^b^	− 0.13	− 0.12	− 0.01	− 0.16	–	0.08	0.00	− 0.19	− 0.28	–	0.14	0.37*	0.03	− 0.01	–

*PaedS* Paediatric Self-Stigmatization Scale, *CGAS* Children’s Global Assessment Scale

*p < 0.05; **p < 0.001

Finally, we performed a sensitivity analysis whereby we examined whether self-reported quality of life or parental reported total SDQ scores were significant predictors of the improvements in stigmatisation and functioning observed at the follow-up. Results of the linear regression models suggested that neither total SDQ scores nor self-reported quality of life were significant predictors of the latent score differences of the main outcome measures.

## Discussion

To our knowledge, this is the first study to prospectively investigate the relationship between the experience of stigma and the type of mental health treatment (inpatient vs. outpatient) in children, as well as between stigma and children’s global functioning.

Our first hypothesis, that there would be no difference in stigma between children treated for mental health difficulties within inpatient vs. outpatient settings, was to a large extent supported. At baseline, there were no differences in stigma scores for any of the stigma subscales, other than personal rejection, which was greater for inpatients. At follow-up, the only statistically significant difference was in the self-stigma subscale score, with children treated as outpatients experiencing less self-stigma. It should be noted, however, that neither of the treatment types were associated with increasing stigma in the course of the study. Indeed, a statistically significant reduction in stigma was observed in societal devaluation, personal rejection and secrecy subscales for the inpatients, and self-stigma and secrecy subscales for outpatients (Table 2). As such, the difference in self-stigma between the groups at follow-up was not due to self-stigma increasing for inpatients but was as the result of a greater reduction in self-stigma for those treated as outpatients. Noticeably, our results also suggest presence of inter-individual variability in improvement between the two assessments for both inpatients and outpatients with respect to the various aspects of stigma examined. Stated differently, we found that there are significant between-children differences in the rate of reduction of stigma within both groups. Future research should aim to identify characteristics that can enhance or hinder reduction in order to inform targeted interventions aiming to reduce stigma associated with mental illness.

These findings are not out of line with adult mental health literature. Although a greater number of psychiatric admissions in adults has been associated with increased stigma in one study [28], and a similar treatment “dose” effect was also found in the comparison of full-time with part-time hospitalised patients [37] other studies have not identified the same trend [38, 39]. Swital and colleagues [38] also found no difference in the experience of stigma between inpatients and outpatients treated for schizophrenia, whilst Szcześniak and colleagues [39], using a measure of internalised stigma, found no difference in stigma between inpatients and those treated as outpatients on daily units. Factors increasing stigma during hospitalisation may include treatment in larger wards and with more individualised programmes [40], although this has not been invariably reported [41]. The relatively small size of our inpatient unit may have played a role in children feeling more integrated and therefore not experiencing an increase in stigmatisation due to this factor. Furthermore, stigma associated with inpatient admission may be a result of discrimination experienced after discharge, rather than a direct result of admission. Unfortunately, no studies including adult patients have used a longitudinal design to allow comparison with our follow-up findings.

Our second hypothesis, that stigma is linked to functional impairment, with improvements in function associated with reduced stigma, was not supported. Global functioning, as measured by the CGAS, improved for both groups over the study period, which is concordant with previous research [42]. This improvement was greatest for the children treated as inpatients, who had lower functioning at baseline, to the point where there was no difference between the groups at follow-up. Whilst a reduction was also observed for many of the stigma subscales, the partial correlation analysis showed that the rate of change in functioning was not associated with the rate of change in any of the stigma subscales when the two treatment groups were analysed together. When analysed separately, the change in personal rejection subscale score for the outpatient group was positively associated with the change in CGAS. This correlation is reflective of the fact that that a reduction in personal rejection is associated with an improvement in functioning, albeit this was evident among outpatients only.

The lack of an association between the experience of stigma and functional improvement would warrant further investigation both in children and young people and in adults with mental health difficulties. The importance of further exploring the relationship between stigma and functioning lies within the possibility to reduce stigma through optimization of interventions to maximise functional outcomes in addition to public campaigns targeting it directly. Indeed, this association was found in a recent study of adults with first episode psychosis [43], where patients were stratified into three groups, depending on whether they perceived stigma at either baseline or one-year follow-up, both timepoints, or neither. Those perceiving no stigma had higher levels of global functioning than those experiencing stigma at both timepoints. However, the differences in methodology between this study and ours would not allow for direct comparisons. Firstly, our study used change in stigma, rather than its presence or absence as the variable of interest. Furthermore, Simonsen and colleagues [43] used a fixed 1-year follow-up period, whereas we employed a clinically informed endpoint for the children treated as inpatients (discharge). Importantly, they found that the sustained experience of stigma may be associated with poorer global functioning, although the direction of causality cannot be determined.

Finally, to better characterise our cohort, measures of symptom severity and quality of life were used in addition to clinician assessed global functioning. As expected, inpatients demonstrated clear improvements in scores for all reported SDQ subscales as well as quality of life, over the course of treatment. On the other hand, for those treated as outpatients, the SDQ subscales and quality of life scores were relatively static over the same period of time. Given the intensity of inpatient work this is not an unexpected finding and demonstrates that inpatient input can be very helpful for children needing it without significantly affecting their experience of stigmatisation.

This study has several strengths. It is the first study to directly compare stigma between children treated inpatients and outpatients from the perspective of the child. Furthermore, by using a prospective design, we were able to explore the impact of treatment on stigma over time. A recently validated, quantitative measure of stigma from the child’s perspective was used alongside established clinician rated global functioning measures and parental-reported symptom scales. Within the sample, participants were well matched for diagnosis, age and sex. A difference in follow-up time between the two groups was observed, however, statistical adjustment was used to mitigate this. The most significant limitation of the study was the relatively small sample size due to the rare need for children to be admitted in hospital and the relatively long admissions of these children determined by clinical need in the UK system. However, this also allowed the prospective exploration of stigma over time during these admissions which would not have been otherwise possible. Using mixed methods designs, extending the follow-up period including post-discharge from an inpatient setting, and expanding to other cultural contexts in future studies could advance further the understanding of stigma experiences and processes in this age group.

## Conclusion

We identified that the experience of stigma associated with mental health treatment in children was to a large extent not related with the setting (inpatient vs. outpatient) where this treatment takes place and seems to reduce in the short-term course of it. It also does not seem to be related to functional improvements resulting from professional input. This stigma is not well understood and remains an important area for future research. Understanding its nature, extent, and associations is likely to facilitate the development of effective strategies to tackle it and increase access to and compliance with treatment. This way, the burden associated with mental health difficulties in children will be alleviated as much as possible and evidence-based interventions will be appropriately implemented, positively affecting the course and prognosis of these difficulties.
